# Challenging delivery of VLHL NS plasminogen activator inhibitor-1 by osmotic pumps in diabetic mouse: A case report

**DOI:** 10.3892/etm.2012.639

**Published:** 2012-07-17

**Authors:** JERZY JANKUN

**Affiliations:** Urology Research Center, Department of Urology, Health Science Campus, University of Toledo, Toledo, OH, USA; Department of Clinical Nutrition, Medical University of Gdańsk, Gdańsk, Poland; Protein Research Chair, Biochemistry Department, College of Sciences, King Saud University, Riyadh 11451, Kingdom of Saudi Arabia

**Keywords:** osmotic pump, diabetes, plasminogen activator inhibitor-1

## Abstract

ALZET^®^ osmotic pumps are implantable devices used in animals for the continuous infusion of drugs or proteins at controlled rates from 1 day to 4 weeks. Pumps have been used successfully in a number of studies on the effects of controlled delivery of a wide range of experimental agents, independent of their properties. In the present study, use of these pumps was made in mice with diabetic nephropathy. Plasminogen activator inhibitor-1 (PAI-1) mediates diabetic nephropathy, which is characterized by the excessive accumulation of extracellular matrix (ECM) in the kidney. Disproportionate PAI-1 inactivates tissue plasminogen activator, which is one of the proteolytic enzymes in a cascade responsible for ECM remodeling in the kidney. The decrease of PAI-1 in the kidney has been shown to arrest the progression of nephropathy in experimental animals. This was achieved using inactive PAI-1R which increased the clearance of wild-type PAI-1 in order to protect net proteolytic activity and ECM clearance. However, this protein has a brief half-life *in vivo*, therefore, high and frequent doses are required. Thus, VLHL NS PAI-1 protein with a long half-life of over 700 h (Gln197Cys, Gly355Cys) inactivated by single point mutation (Arg369Ala) was used. Following the sacrifice of animals the tips of the flow moderators of the osmotic pumps in the treated animals were found to be clogged. In addition, from each pump from the treatment group, but not controls, we collected 50–150 *μ*l of clear liquid containing VLHL NS PAI-1, cellular and serum proteins suggesting early pump sealing by cellular material. In conclusion, despite encouraging results obtained for the PAI-1R protein, the method of VLHL PAI-1 delivery should be ameliorated.

## Introduction

ALZET^®^ osmotic pumps are implantable pumps used for research in mice and other animals for the continuous infusion of drugs or proteins at controlled rates from 1 day to 4 weeks, without the need for external handling. These pumps are used for systemic administration by implantation subcutaneously or intraperitoneally. Pumps have previously been used in a number of studies on the effects of controlled delivery of a wide range of experimental agents, including addictive drugs, steroids, chemotherapeutic drugs, hormones, and antibodies or other proteins. It is extremely important that compounds of any molecular conformation are delivered predictably at controlled rates, independent of their properties (1–4). In the present study, the pumps were used to deliver plasminogen activator inhibitor-1 (PAI-1) to successfully reduce tumor size in SCID mouse in the past (5).

In this study, ALZET osmotic pumps were used on mice with diabetic nephropathy. Various factors have been suggested in the pathogenesis of diabetic nephropathy, including an increased PAI-1 level in plasma. PAI-1 mediates diabetic nephropathy, which is characterized by excessive accumulation of extracellular matrix (ECM) in the kidney. Excessive PAI-1 inactivates tissue plasminogen activator, which is one of the proteolytic enzymes in a cascade responsible for ECM remodeling in the kidney. A decrease of PAI-1 in the kidney has been shown to arrest the progression of nephropathy in experimental animals. This decrease was achieved using inactive PAI-1R which increased clearance of wild-type PAI-1 in order to protect net proteolytic activity and ECM clearance (6,7). However, this protein has a brief half-life *in vivo,* therefore, high and frequent doses are required for it to be effective. VLHL NS PAI-1 (8) with a long half-life of over 700 h (Gln197Cys, Gly355Cys) inactivated by single point mutation (Arg369Ala) was therefore used (9). We hypothesized that this protein is likely to prevent nephropathy when used in the early stages of diabetes and arrest its progression in advanced stages of this disease.

VLHL NS PAI-1 was loaded into osmotic pumps to deliver protein over a 2-week period in Dock7^m^ +/+ Lepr^db^ diabetic mice to observe whether it had any effects on diabetes. All pumps containing VLHL NS PAI-1 were found to be clogged and the majority of the buffer with the active ingredient remained within the pumps while the control pumps contained little, if any, buffer. Analyses of proteins in the pumps suggests that the pumps were clogged by cellular material early in the experiment.

## Materials and methods

### VLHL NS PAI-1

VLHL NS PAI-1 was expressed and purified as reported previously (9). VLHL NS PAI-1 was in an active conformation with traces of VLHL NS PAI-1 in latent conformation. The purity of protein was determined as high as +95% by densitometry (9).

### Animals

Animals were purchased from The Jackson Laboratory (TJL; Bar Harbor, ME, USA) and maintained according to TJL recommendations. Mice homozygous for the diabetes spontaneous mutation (Lepr^db^), strain name BKS. Cg-Dock7^m^ +/+ Lepr^db^/J (db/db), become obese at 3–4 weeks of age. Elevation of plasma insulin begins at 10–14 days and elevation of blood sugar at 4–8 weeks. Homozygous mutant mice are polyphagic, polydipsic and polyuric. The severity of disease on this genetic background leads to an uncontrolled rise in blood sugar, severe depletion of the insulin-producing β-cells of the pancreatic islets and death by 10 months of age. Exogenous insulin fails to control blood glucose levels and gluconeogenic enzyme activity increases. Peripheral neuropathy and myocardial disease are observed and wound healing is delayed. An increased amount of PAI-1 in kidney and in animal serum was also detected (6,7).

The db/db mouse is the model that develops abnormalities in renal morphology and function that parallel those in human nephropathy of type 2 diabetes (6). Following uninephrectomy at the 8th week of age, a greatly accelerated development and progression of diabetic nephropathy was reported. Increased levels of glucose were observed between weeks 8 and 20 with an increase of PAI-1 in kidney tissue. Uninephrectomized diabetic db/db mice developed nephropathy by 20 weeks of age, manifested by expansion of the mesangium and significant albuminuria (6,7).

At 20 weeks, animals had an intra-abdominal osmotic pump implanted to administer the buffer (control group) or VLHL NS PAI-1 (treatment group). The pumps were purchased from Durect Corporation ALZET Osmotic Pumps (Cupertino, CA, USA). The treatment group mice (n=6) were implanted with ALZET pump #2002 filled with 200 *μ*l of VLHL NS PAI-1 (350 *μ*g of protein). These pumps are designed to deliver 0.5 *μ*l/h over 16 days at a total of ∼200 *μ*l. Similarly, the control group mice (n=6) were implanted with ALZET pump #1002 filled with 100 *μ*l of PBS. These pumps are designed to deliver 0.25 *μ*l/h over 16 days for a total of ∼100 *μ*l. At 22 weeks of age (14 weeks post-uninephrectomy), the two groups of animals were placed in metabolic cages for 24 h to collect 24-h urine samples. At the end of 24 h, blood samples were collected via cardiac puncture under anaesthesia and sacrificed via exsanguination.

### Mass spectrometry-based proteomic analysis

The mass spectrometry-based proteomic analysis was performed at the Proteomics Resource Facility, Department of Pathology (Ann Arbor, MI, USA) using multidimensional proteomic identification technology. The data were searched against a mouse database appended with human PAI-1 (UniProt accession, #P05121).

## Results and discussion

**Figure 1:**
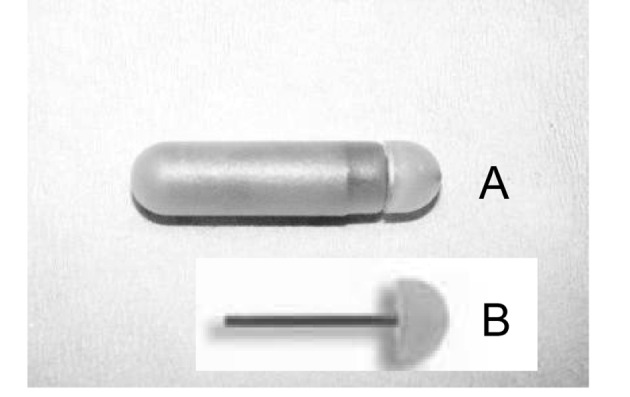
(A) Fully assembled ALZET^®^ osmotic pump; (B) flow modulator.

**Figure 2:**
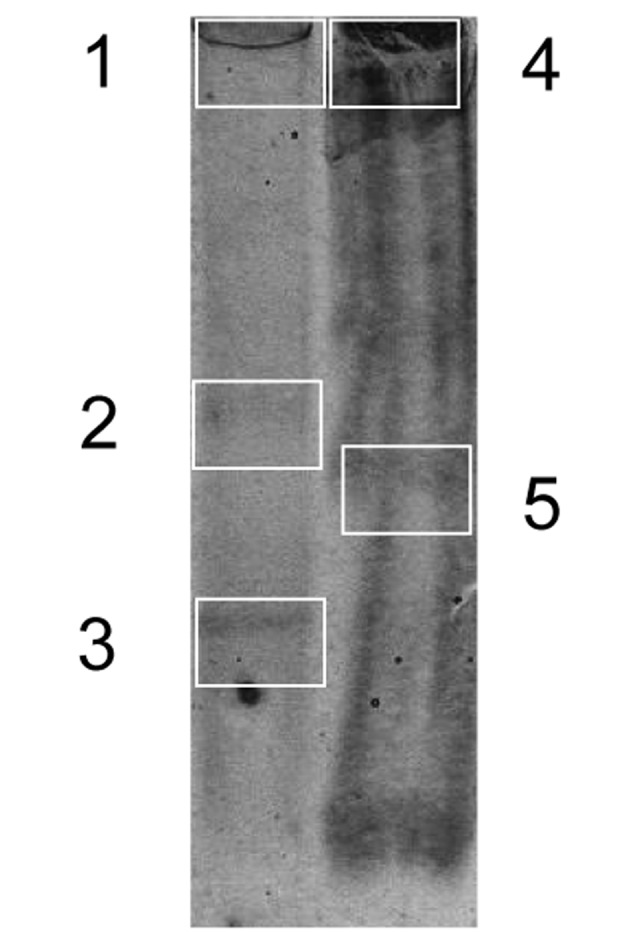
SDS-PAGE gel of liquid collected from two different pumps and approximate position of cuts analyzed by mass spectrometry. Each of the gel cuts (1–5) was analyzed separately by mass spectrometry.

At the end of the experiments, the mice showed signs of severe obesity and severe diabetes (data not shown). After sacrifice it was observed that the tips of the flow moderator of osmotic pumps in the treated animals were clogged by yellow cell material. The control group was free of obstructing material. On close examination it was noted that the tip plug was 1- to 3-mm long and difficult to remove from the flow modulator (Fig. 1). In addition, in each pump from the treatment group we collected 50–150 *μ*l of clear liquid. Almost no liquid remained in the pumps of the control group. The collected liquid from two pumps of the treatment group was analysed by SDS-PAGE electrophoresis. As shown in Fig. 2, numerous protein bands were detected. Randomly chosen samples were cut from the SDS-PAGE gel and sent for protein identification. Numerous proteins were detected in various locations.

The dominant protein was VLHL NS PAI-1 identified as wild-type human PAI-1 (Table I). This protein was detected in all bands suggesting multiple complexes with other proteins. It is also proof that VLHL NS PAI-1 remains in the pump, i.e., it was delivered as intended and the pumps were blocked early. The other elevated proteins were identified as tubulin, serpina 1a, serpina 1d and serum albumin. These, and less abundant proteins, were of circulatory and cellular origin (Table I). ALZET^®^ pumps operate at an osmotic pressure difference between a compartment within the pump and the tissue environment in which the pump is implanted may reach at least 0.5 atm (10). Thus, the stopper was relatively rigid and could be made from adipose tissue abundant in the intra-abdominal cavity of diabetic mice. Our initial hypothesis was that VLHL NS PAI-1 attracted the cells into the tip of the flow modulator of the osmotic pump. Plasminogen activator inhibitor has been reported to be a motility factor (11,12) and adhesion factor, however, inactive PAI-1 [P14 mutant (Thr333Arg)] failed to enhance adhesion (13). Thus, the phenomenon remains unexplained, and a literature search failed to yield any studies on similar incidents. Additionally, the manufacturer was not aware of such incidents.

## Figures and Tables

**Table I:** Proteins detected in the SDS-PAGE gel.

UniProt #	Protein name	Probability	Line on gel	Relative concentration^a^
A0AUV1	Histone H2A	0.999	1,2	
A1E281	β-actin	0.999	2	
A2AL35	Gelsolin, isoform CRA_b	0.999	2	
A2CG44	MAD homolog 3 (*Drosophila*)	0.999	1,2	
A5JUZ1	Ubiqutin subunit 1	0.994	1,2	
A8DUK0	Hbbt1	0.999	1,2	
A8DUV3	α-globin	0.999	2	
B2RSN3	Tubulin, β 2B	0.999	1,4,5	High
B7U582	Heat shock protein 70-2	0.999	1,2,3	
B7ZNJ1	Fn1 protein	0.999	1	
B8JJM3	Complement factor B	0.999	2	
D3YTY9	Putative uncharacterized protein Kng1	0.999	3	
D3YV43	Putative uncharacterized protein Rps3	0.999	4	
D3YVC1	Putative uncharacterized protein Rps2	0.995	4	
D3YVF4	Putative uncharacterized protein Rps14	0.999	4	
D3YW44	Putative uncharacterized protein Gm5121	0.999	4	
D3YW52	Putative uncharacterized protein Pzp	0.999	1,2	High
D3YYR8	Putative uncharacterized protein Trf	0.999	2,3	
D3Z0D8	Putative uncharacterized protein Rrm2	0.999	4	
D3Z3P6	Putative uncharacterized protein ENSMUSP00000032206	0.994	1	
D3Z451	Putative uncharacterized protein Gm4931	0.999	3	
D3Z6U8	Putative uncharacterized protein Fmr1	0.999	4	
**P05121**	**HUMAN plasminogen activator inhibitor 1**	**0.999**	**1,2,3,4,5**	**Very high**
Q14AS7	Serine (or cysteine) peptidase inhibitor, clade A, member 3C	0.999	3	
Q2F3J4	Truncated ceruloplasmin	0.999	2	
Q3KQQ4	Serpina1a protein	0.999	3	High
Q3TGR2	Putative uncharacterized protein	0.999	1	
Q3TII3	Elongation factor 1-α	0.999	1,4	
Q3TX45	Gene name, Apoe; putative uncharacterized protein	0.999	1	
Q3U9U3	Gene name, Tubb6; putative uncharacterized protein	0.999	1,4,5	High 4,5
Q3UBS3	Gene name, Hp; putative uncharacterized protein	0.999	2	
Q3UID0	Gene name, Smarcc2; putative uncharacterized protein	0.999	1	
Q3UKP2	Gene name, Hpx; putative uncharacterized protein	0.999	2,3	High 3
Q3UKX6	Gene name, Apoa2; putative uncharacterized protein	0.999	1,2	
Q3V2E0	Gene name, Try5; putative uncharacterized protein	0.999	3	
Q3V2G1	Gene name, Apoa1; putative uncharacterized protein	0.999	1,2	High 2
Q543J5	Serine (or cysteine) peptidase inhibitor, antithrombin	0.999	3	Very high
Q546G4	Serum albumin	0.999	1,2,3,4,5	
Q58E61	Igh protein	0.999	1	
Q5FW91	Tubulin, α 3B	0.999	1,4,5	High
Q5M9K1	Transthyretin	0.999	2,3	
Q65ZL8	VH7183-DSP2-JH3-CH1 protein	0.999	1	
Q80XP1	Complement component 3	0.999	2	
Q810I7	Apoa4 protein	0.999	1,2	
Q8C4B1	Gene name, Larp1b; putative uncharacterized protein	0.999	1	
Q8K051	Trip12 protein	0.999	1	
Q8VC41	Serpina1d protein	0.999	2,3	High 3
Q9CZQ0	Gene name, Nudt21; putative uncharacterized protein	0.999	2,3	
Q9JHV2	Lectin-related NK cell receptor LY49T	0.992	2	
Q9Z1R9	Trypsinogen 16	0.994	1,2	

Bold represents human protein VLHL NS PAI-1 added to pumps.

a Relative concentration shows number of different polypeptides of individual protein detected by mass spectrometry.
